# Leveraging the role of the microbiome in endometriosis: novel non-invasive and therapeutic approaches

**DOI:** 10.3389/fimmu.2025.1631522

**Published:** 2025-09-16

**Authors:** Eleni Andria Kalopedis, Amine Zorgani, Dmitry A. Zinovkin, Muruj Barri, C. David Wood, Md Zahidul I. Pranjol

**Affiliations:** ^1^ Department of Biochemistry, School of Life Sciences, University of Sussex, Brighton, United Kingdom; ^2^ SwipeBiome, Loos, France; ^3^ Department of Pathology, Gomel State Medical University, Gomel, Belarus

**Keywords:** endometriosis, gut microbiome, dysbiosis, immunomodulation, microbiota-based therapy, estroblome, biomarkers, probiotics

## Abstract

Endometriosis (EMS) is an oestrogen-dependent condition characterised by ectopic endometrial-like tissue growth with a chronic and inflammatory nature leading to severe symptoms and reduced quality of life. Emerging evidence implicates gut microbiome dysbiosis in EMS pathogenesis, driving chronic inflammation, immune dysfunction, and altered bacterial taxa within patient gut microbiome. This review examines the intricate relationship between gut dysbiosis and EMS, with a focus on immunomodulatory mechanisms and the downstream consequences of the bacterial contamination theory. It evaluates recent findings regarding microbial imbalances and microbial diversity, pinpointing gaps in current research that mandate further understanding. For example, while microbial markers like *Lactobacillus* depletion and elevated *Escherichia coli* have been observed in patients, their diagnostic potential remains poorly defined. Additionally, it addresses the broader implications of EMS, including its physical, mental and healthcare burdens. Simultaneously, critiquing current drawbacks in diagnostic and therapeutic strategies such as their invasiveness and limited efficacy. The review further evaluates novel microbiome-based strategies namely *Lactobacillus*-based probiotics and faecal microbiota transplantation (FMT), assessing their potential in modulating immune responses and alleviating EMS symptoms while considering associated challenges. Lastly, it highlights the emerging role of metabolomics in identifying non-invasive and diagnostic biomarkers like short-chain fatty acids (SCFAs), implicated in the interplay between microbial metabolites and immune signalling pathways in EMS.

## 1 Introduction to the epidemiology, pathophysiology and management of endometriosis

Endometriosis is a chronic, inflammatory, oestrogen-dependent gynaecological condition characterised by the ectopic growth of endometrial-like tissue outside the uterine cavity, leading to a range of debilitating symptoms (1). The term “Endometriosis” is derived from Greek: “endo” (within), “metra” (uterus), and “osis” (disease), with pelvic pain as the primary symptom. Risk factors include a shorter menstrual cycle, alcohol use, caffeine intake and earlier age at menarche (2). The condition is thought to affect approximately 10% of people assigned female at birth (3), with 6 - 10% of individuals of childbearing age affected (4) however, prevalence may vary depending on the population studied. Regardless, EMS remains a significant cause of infertility and reduced quality of life (5, 6) for an estimated 176 million women globally (7).

The most widely recognised theory for EMS development is the ‘Retrograde Menstruation Theory’, where endometrial fragments shed during menstruation flow back through the fallopian tubes. These fragments can then implant in the pelvis (ovaries, fallopian tubes, peritoneal surfaces, bowel, bladder), proliferating into invasive lesions that bleed and grow in a manner similar to the uterine lining. This process results in the development of adhesions, fibrosis, and ultimately localised inflammation (8–10). While this theory provides some groundwork, it fails to explain the clinical heterogeneity of the disease or why many women experience retrograde menstruation without developing EMS. This indicates that other underlying mechanisms are implicated, with the gut-immune axis and the balance of the microbiome gaining significant awareness. Dysbiosis in the gut can drive systemic inflammation and immune aberrations, creating an environment conducive to the survival and proliferation of ectopic endometrial cells. This critically influences the susceptibility to and progression of endometriosis beyond the initial cellular translocation.

Beyond immune dysregulation, imbalances and alterations in gut microbiota such as an altered *Bacillota/Bacteroidota* ratio have also been implicated (11). This dysbiosis is postulated to compromise pelvic stability by disrupting local immunomodulation, leading to a cycle of inflammation, pain, and tissue damage. The resulting immune and microbial imbalance is at the core of the diverse symptomatology of EMS, including dysmenorrhea, dyspareunia, infertility and chronic pelvic pain (12, 13). Recognising and leveraging the immunomodulatory role of the gut and vaginal microbiome should be prioritised due to the vast drawbacks of current therapeutic and diagnostic approaches such as side effects, high recurrence rates and limited detection of lesions in asymptomatic women (4).

## 2 The role of gut microbiome in driving dysbiosis in endometriosis

### 2.1 Role of the gut microbiome in health and disease

The gut microbiome comprises all microorganisms residing in the gastrointestinal tract, including their genes and metabolites, within a specific anatomical site. In contrast, the microbiota refers solely to the community of microorganisms such as bacteria, viruses, fungi, archaea, and protozoa (14). Amid the body’s microbiomes, the gut bacteria is the most extensively studied due to its critical roles in nutrient absorption, synthesis, immune system development, mucosal health, and host defence (15). Despite their smaller size, bacterial cells are as numerous as human cells, with their microbiome encoding over three million genes; 150 times more than the human genome (16). The importance of the gut in health was recognised as early as 400 B.C., when Hippocrates stated, “Death sits in the bowels” (17). Beyond gastrointestinal function, a fair amount of research is discussing the role of microbiome as a major regulator and biomarker for numerous inflammatory and proliferative diseases (18–20). For example, Long et al. (21) identified 11 microbiota-related causal links to cancers, including breast cancer (22). Research continues to explore the microbiome’s vast genetic potential in modulating immune responses, nutrient metabolism, neuromodulation, and barrier integrity.

The gut microbiome exists in two primary states. The *eubiotic* state supports homeostasis through immune and endocrine regulation, nutrient absorption, and protection against pathogens (23). Conversely, *dysbiosis*, marked by alterations in microbiota composition, compromises these and is linked to impaired intestinal barrier function, inflammation, and diseases such as obesity, hypertension, cardiovascular and neurological disorders, diabetes, and inflammatory bowel disease (24–26). Qin et al. (27) metagenomically analysed 650 bacterial and archaeal genomes to identify a ‘common core’ microbiome in eubiotic and dysbiotic environments. Eubiotic microbiota were dominated by *Bacillota* and *Bacteroidota* (>90%), with low levels of *Pseudomonadota*. By contrast, dysbiotic microbiota, displayed an altered *Firmicutes/Bacteroidetes* (F/B) ratio and changes in *Lactobacillus* abundance. Investigations into the transition to a dysbiotic state have primarily employed measures of alpha diversity, taxa abundance, and the *Bacillota/Bacteroidota* ratio.

**Figure 1:**
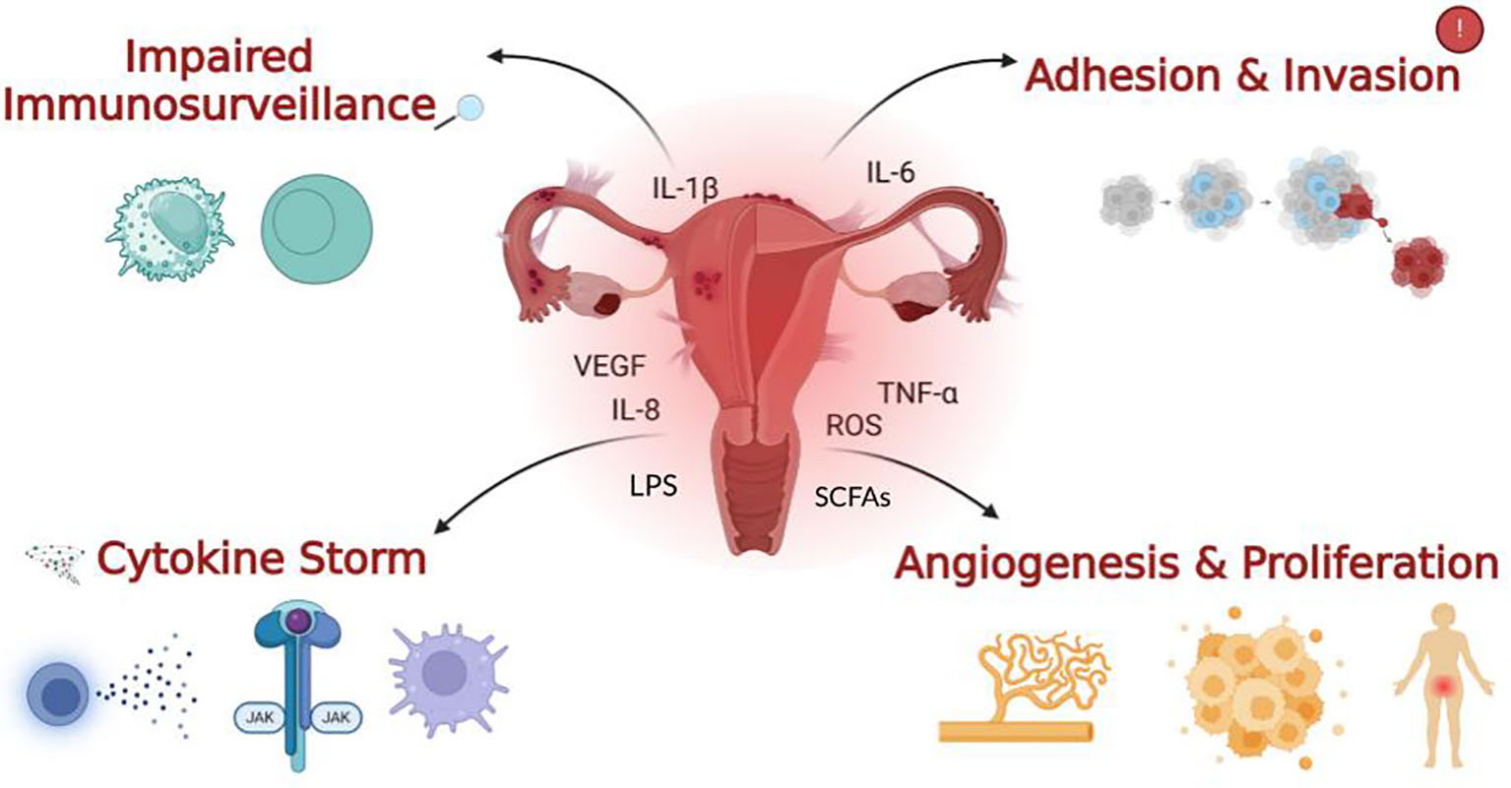
The four main outcomes of immune dysregulation in endometriosis. Impaired immune surveillance is characterised by a reduction in natural killer (NK) cell activity observed in EMS patients. A cytokine storm reflects the excessive recruitment and over-activation of pro-inflammatory cytokines and immune cells, which heavily contribute to chronic and severe inflammation. Within the context of gut dysbiosis, an increase in gut permeability can lead to elevated levels of pro-inflammatory bacterial metabolites like lipopolysaccharide (LPS) entering systemic circulation, exacerbating this immune dysregulation. Contrarily, a reduction in beneficial Short-Chain Fatty Acids (SCFAs), produced by a healthy gut microbiota, diminishes their anti-inflammatory and immune-modulating effects, further contributing to the inflammatory cascade. The immune cells, cytokines and pro-inflammatory factors depicted in the centre of the image, along with adhesion, invasion, angiogenesis and proliferation on the right, arise as downstream effects of diminished immune surveillance and increased inflammation as illustrated on the left. Together, these processes promote lesion survival and further exacerbate the inflammatory nature of the condition playing a major role in the pathogenesis of EMS.

Expanding the scope beyond the extensively studied gut microbiome, the vaginal microbial ecosystem represents a critical, yet often underappreciated, facet of women’s health, especially relevant in the context of EMS. The vaginal microbiome is a complex community of microorganisms, with its composition significantly influencing local immunity and susceptibility to gynaecological conditions (28) (**Figure 1**). In healthy women, the vaginal environment is usually dominated by species of the genus *Lactobacillus*, which play a pivotal role in maintaining homeostasis (29). Focusing more on the maintenance and modulation of immunity, recent evidence continues to elucidate the intricate relationship between the vaginal microbiome and inflammation. For instance, Yichan et al.’s (30) study on Chinese women demonstrated a negative correlation between the presence of *Lactobacillus crispatus* and *Lactobacillus iners* and pro-inflammatory cytokines IL - 1α and IL - 1β, while conversely, non-*Lactobacillus* species like *Gardnerella vaginalis* and *Escherichia coli* showed positive associations (30). This aligns with another recent finding which highlights that vaginal dysbiosis, marked by a reduction in *Lactobacillus* dominance and increased microbial diversity, is linked to an elevated risk of adverse genital tract diseases, pregnancy complications and can trigger pro-inflammatory responses by impairing the vaginal mucosal barrier (31). Given the anatomical proximity to the pelvic cavity and the potential for systemic immune modulation, understanding the functions and dynamics of the vaginal microbiome is increasingly recognised as crucial for a holistic understanding of gynaecological health and conditions such as EMS.

### 2.2 Microbial alpha diversity alterations in endometriosis as a consequence of dysbiosis

**Figure 2:**
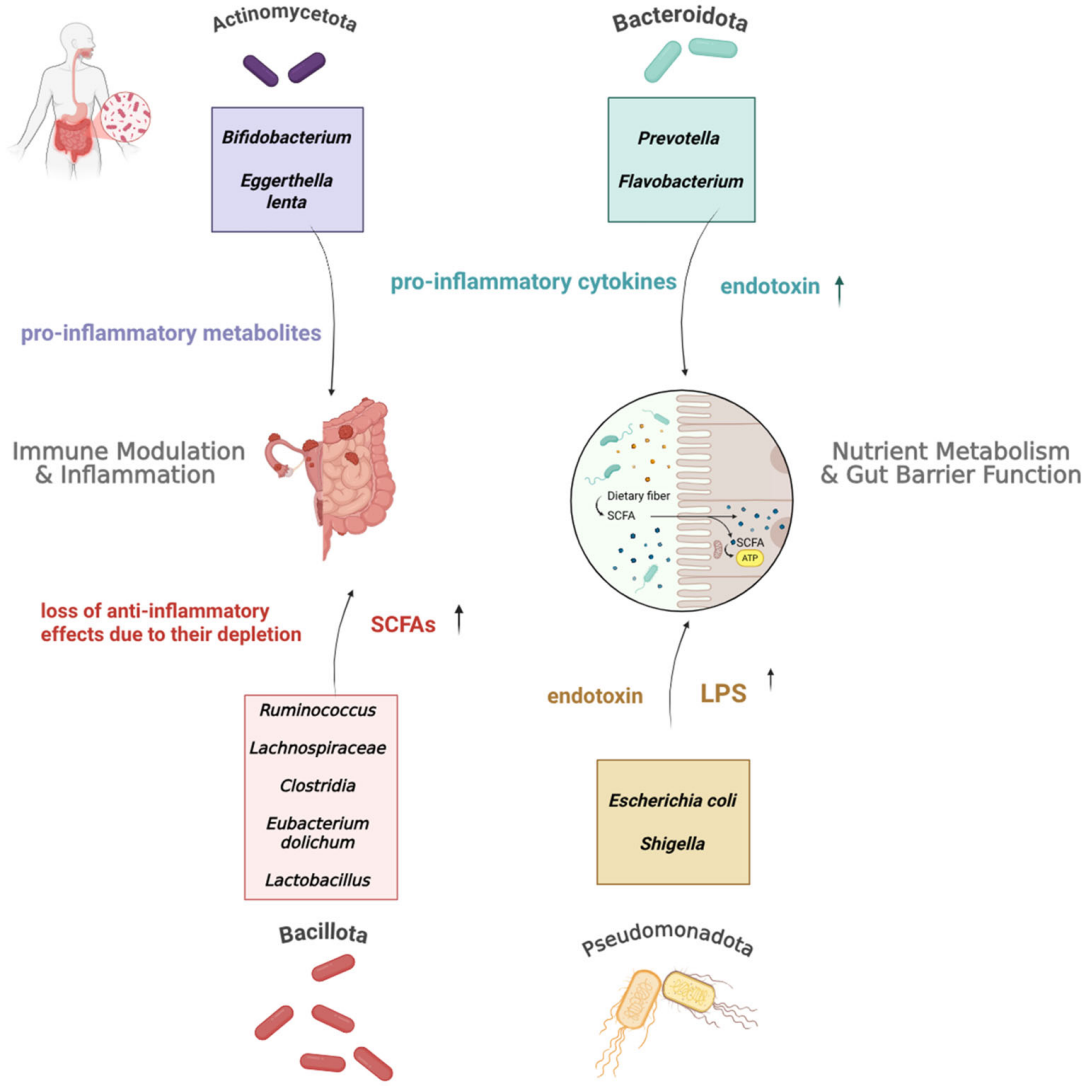
Interplay Between the Four Main Gut Microbiome Phyla, their Respective Taxa, and Immune Dysregulation in Endometriosis. As highlighted in the studies discussed above, these phyla and taxa exhibit altered abundances in EMS patients. This network illustrates their dysregulated immunomodulatory roles within the context of gut dysbiosis and emphasises their biological contribution to the progression of the condition.

Numerous studies highlight how altered microbial diversity and populations are present in EMS patients, however the nature of these changes is still unclear (**Figure 2**). Diversity alterations were represented by Shannon (represents both richness and evenness) and Simpson (focuses on evenness) biodiversity measures (Chen, 2021). Many studies reported reduced microbial richness, for instance, a study conducted by Lin et al. (32), observed reduced Shannon and Simpson measures within faecal samples from EMS patients; 10.5% (*p* = 0.006) and 5.7% (*p* = 0.013) decreases, respectively (32). In concordance, Svensson also saw a marked reduction in the alpha diversity of EMS patients (p = 4.9 × 10^−5^) as did Shan et al. (33, 34). For adults, a less diverse gut microbiome has been linked to reduced production of beneficial metabolites like short-chain fatty acids (SCFAs), potentially leading to impaired mucosal immunity and contributing to immune dysregulation and chronic inflammation in EMS (35). To substantiate this, a study on a murine model found that mice with endometriosis had significantly lower concentrations of SCFAs, such as n-butyrate, compared to healthy controls. This research further demonstrated that n-butyrate directly inhibited the growth of human endometriotic epithelial and stromal cells *in vitro*, highlighting a direct anti-proliferative effect (36). Additional pioneering evidence from a separate murine model revealed that Fecal Microbiota Transplantation (FMT) from healthy donors elevated the levels of the SCFA acetate in both the gut and ectopic lesions, which in turn activated the JAK1/STAT3 signalling pathway ultimately driving macrophages towards an anti-inflammatory M1 phenotype within the lesions. Conversely, FMT from endometriosis patients with reduced acetate production exacerbated the condition (37). The current discussion primarily centers on SCFAs, but for a wider perspective on other beneficial metabolites, readers are referred to the review by Liu et al. (38). In contrast to these findings in the gut, other research has indicated increased microbial diversity within the endometrial tissue of EMS patients, with statistically significant associations reported between higher bacterial diversity and EMS (39: p = 0.09; 40: p = 0.036).

These inconsistent findings regarding microbial diversity in EMS are likely influenced by several methodological limitations. Notably, studies by Shan et al. (33) and Wessels et al. (39) were constrained by small sample sizes and issues with control group definition. For instance, Shan et al. lacked laparoscopic confirmation to definitively exclude EMS in their controls, while Wessels et al. did not include a healthy control group to establish a baseline endometrial microbiota profile. Furthermore, Svensson et al. (34) highlighted the potential for undiagnosed EMS within their control cohort and the restricted functional insights afforded by 16S rRNA sequencing. While these results appear inconsistent, they may also reflect distinct anatomical niche-specific dynamics driven by the pathogenesis of the disease. It is hypothesised that systemic dysbiosis in the gut, characterised by an overall reduced alpha diversity, could lead to a compromised intestinal barrier. This impaired barrier could then facilitate the translocation of opportunistic bacteria from the gut to the peritoneal cavity and endometrial tissue. This translocation process presents novel microbial species to the local endometriotic environment, resulting in a paradoxical increase in local alpha diversity at the site of the lesions. While methodological limitations inarguably contribute to these discrepancies, a synthesis of the data suggests that a systemic loss of diversity can precipitate a localised increase in pro-inflammatory bacterial diversity. To achieve greater clarity and establish a consensus, future research should prioritise larger, well-controlled, multi-omics studies. These investigations must incorporate comprehensive controls for library preparation and focus on the functional roles of bacteria and their metabolites within peritoneal fluid and endometriotic lesions. This would enable a more complete understanding of their impact on local immunity and inflammation and account for the niche-specific dynamics of microbial diversity in endometriosis.

### 2.3 Specific taxa alterations and their relevance in endometriosis

Several studies have reported concurrent findings regarding dysbiotic shifts in bacterial taxa across the four main gut phyla. For example, Huang et al. (41) and Svensson et al. (34), identified significant abundance reductions in *Bacillota*, including taxa such as *Clostridia* and *Lachnospiraceae*, which are crucial for the hydrolysis of starch and other complex carbohydrates into short-chain fatty acids (SCFAs) like butyrate (42). The reduction of these taxa compromises the gut’s capacity to synthesise these beneficial metabolites, which are vital for regulating inflammation and maintaining intestinal barrier integrity. Additionally, Svensson et al. (34), highlighted the association between an increased abundance of specific genera,such as *Prevotella* and the manifestation of gastrointestinal symptoms, including constipation and bloating which are commonly reported in individuals with endometriosis (34). This suggests that shifts in microbial composition may directly impact gut motility and function, contributing to disease pathophysiology.

Concurrently, an increase in *Actinomycetota*, particularly *Eggerthella lenta*, was observed by Svensson et al. (34). This bacterium has been implicated in the activation of pro-inflammatory Th17 cells and is enriched in other inflammatory conditions, including irritable bowel disease (IBD) (43). Furthermore, studies by Wessels et al. (39) and Ata et al. (44) also reported an enrichment of *Actinomycetota*, specifically in the species *Oxalobacteraceae, Streptococcaceae, Bifidobacterium*, and *Parasutterella*. Ata’s study, in particular, revealed overlapping patterns of dysbiosis across vaginal, cervical, and gut microbial profiles. This multi-site comparison stresses the interconnectedness of microbial communities across the gut and reproductive tract, suggesting that dysbiosis may contribute to immune dysregulation beyond a single anatomical site. Specifically, the enrichment of Actinobacteria has been linked to impaired immunomodulation and the persistence of low-grade inflammation (45, 46), potentially sustaining inflammatory responses within the pelvic environment and promoting disease progression.

Furthermore, the two other major gut microbiome phyla *Pseudomonadota* and *Bacteroidota*, exhibited the highest relative abundance in stool samples, as reported by Huang et al. (41) and Svensson et al. (34), who observed increased levels of *Bacteroidota* and *Parabacteroidota* in EMS patients (34, 41). Enterotoxigenic species within these phyla, such as *Escherichia coli (Pseudomonadota)* and *Bacteroides fragilis* (*Bacteroidota*) are associated with chronic tissue inflammation and the release of carcinogenic and pro-inflammatory mediators (47). Supporting this, additional studies have identified an enrichment of *Pseudomonadota* including *Escherichia* and *Shigella* (44, 48). These taxa are known to disrupt immune homeostasis and activate pro-inflammatory cytokine pathways ultimately, increasing intestinal susceptibility to chronic inflammation (49).

### 2.4 Altered *Bacillota*/*Bacteroidota* ratio and dysbiosis in endometriosis

As previously mentioned, measures of biodiversity, alongside the abundances of specific taxa, serve as key indicators of gut dysbiosis. Another commonly utilised parameter for assessing microbial dysbiosis is the ratio of the two predominant phyla in the gut, *Bacillota* and *Bacteroidota* (27). This ratio has been extensively used in research and has been observed to be elevated in various pathological conditions, including obesity, Alzheimer’s disease, Parkinson’s disease, and type 1 diabetes (50–52). In the context of EMS, evidence also points to an altered *Bacillota/Bacteroidota* ratio indicative of disrupted microbial homeostasis. Shan et al. (33) and Ni et al. (48) reported increases in the ratio amongst EMS patients. Notably, Ni et al. (48) observed a significant two-fold increase of the ratio in an EMS-induced mouse model, strongly suggesting a dysbiotic shift within the gut microbiome. However, the findings are not entirely consistent across studies. For example, Li et al. (53), reported only an incremental increase in the ratio and emphasised fluctuations in specific microbial genera, perhaps reflecting experimental variability or disease progression. These inconsistencies question whether the *Bacillota/Bacteroidota* ratio is a sufficiently specific or sensitive marker of dysbiosis in endometriosis. Further research is necessary to determine whether this metric is robust or if alternative measures of dysbiosis may offer more reliable insights for diagnosis or prognosis.

Overall, there appears to be a consistent shift towards a pro-inflammatory, disrupted gut microbial composition and function in EMS patients. This shift is mainly characterised by an enrichment of proinflammatory *Actinomycetota* genera, a reduction in SCFA-producing *Firmicutes*, and a marked increase in enterotoxic and pathogenic taxa within the *Pseudomonadota* and *Bacteroidota* phyla. However, it’s uncertain whether the observed variability in these findings is attributable to EMS heterogeneity or the influence of external factors such as antibiotic usage, diet, geographical location, age, severity of EMS or hormonal therapies; all of which have a suspected implication in microbial composition (54). Researching these microbial fluctuations further, could help elucidate a mechanism connecting dysbiosis to systemic and localised inflammation but also clarify the biological relevance of the *Bacillota/Bacteroidota* ratio and other aforementioned phyla and taxa. Ultimately, this could provide a plausible link between immunomodulation of gut dysbiosis and EMS pathogenesis. Understanding this in greater detail could strongly support the development of targeted and personalised microbiome-based immunomodulatory interventions.

## 3 Examining the disruption of immunomodulation in endometriosis

### 3.1 Bacterial contamination, immune dysfunction and chronic inflammation

**Figure 3:**
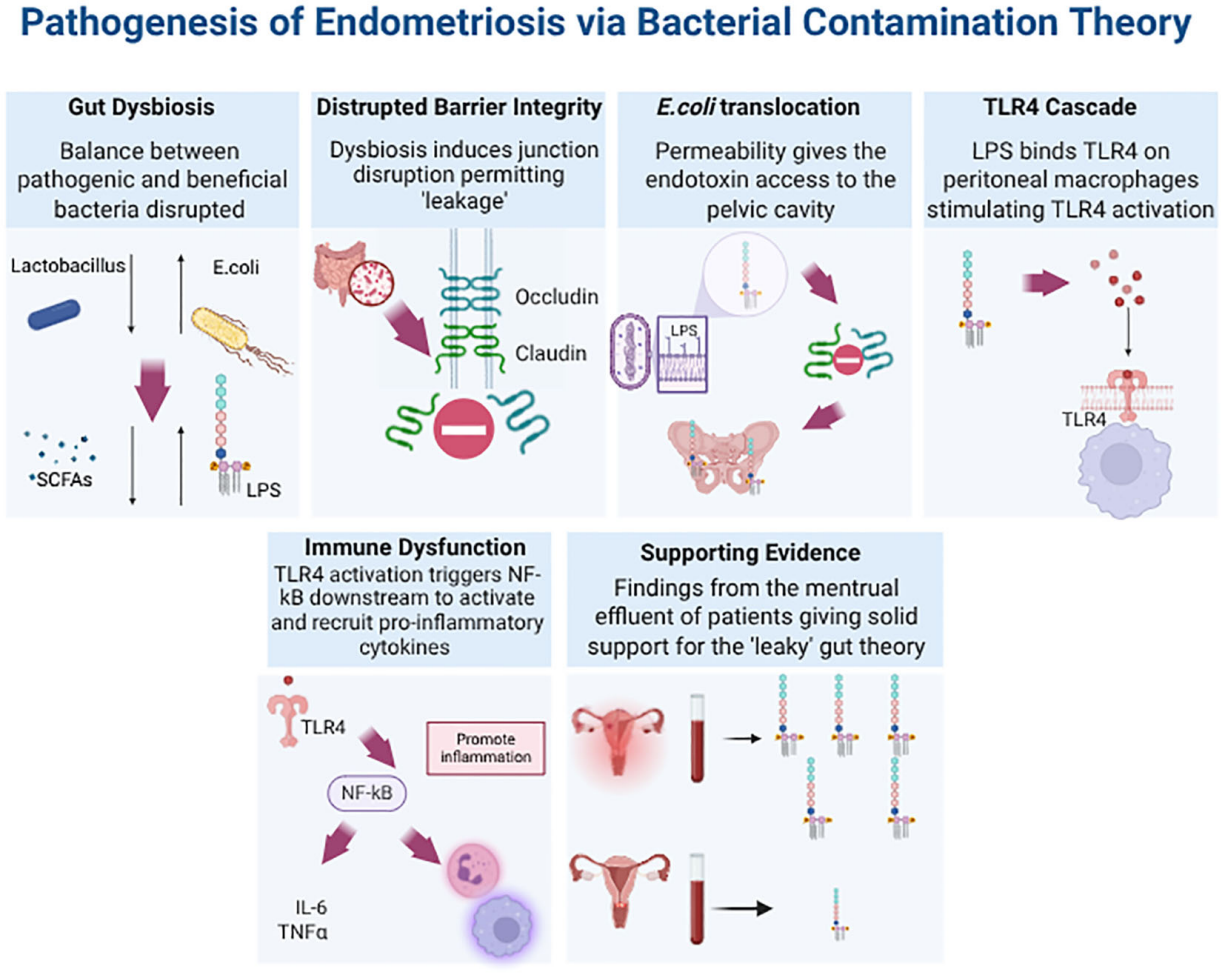
Downstream Effects of Disrupted Epithelial Barrier Integrity Due to Gut Dysbiosis in Endometriosis. The figure illustrates the cascade of signalling events, collectively known as the “Bacterial Contamination Theory.” This theory provides a mechanism explaining how gut dysbiosis in EMS leads to increased concentrations of endotoxins, such as lipopolysaccharide (LPS), in the menstrual effluent of patients. Furthermore, it links the presence of LPS to immune dysregulation and inflammation. In the bottom right corner, a spider diagram summarises how further research into the cellular and molecular components of this signalling cascade could aid in the development of microbiome-based diagnostics and therapeutics.

Emerging evidence suggests a significant association between gut microbiome dysbiosis and immune system dysregulation, contributing to the chronic inflammation that is the hallmark of endometriosis. Khan’s theory highlights how disruptions in gastrointestinal tract maintenance, mucosal integrity, and barrier function promote intestinal permeability, leakage of metabolites, and inflammatory changes (**Figure 3**) (9, 55, 56). In a series of studies, Khan explored the role of lipopolysaccharide (LPS), a bacterial cell wall endotoxin, in initiating and propagating endometriosis when present in the intrauterine environment (57, 58). The research revealed that LPS concentrations in the menstrual fluid of patients with endometriosis were four to six times higher than in controls. Specifically, menstrual fluid endotoxin levels averaged 285.5 ± 64.5 pg/mL in patients compared to 114.9 ± 17.0 pg/mL in controls (*p* < 0.01). Furthermore, menstrual blood samples from patients with endometriosis were highly contaminated with Gram-negative *Pseudomonadota*, such as *Escherichia coli*, with a median concentration of 4.5 Log10 CFU/mL (IQR 1.4 – 7.2), compared to 1.2 Log10 CFU/mL (IQR 0.8 – 1.9) in controls (*p* < 0.01) (58). This observation is supported by evidence that a compromised intestinal barrier may facilitate the translocation of *E. coli* from the gut to the pelvic cavity via enterocytes (44). The inflammatory cascade is pivotal in understanding this process. Supporting this link between gut and pelvic dysbiosis, Ata et al. (44) previously demonstrated that patients with moderate-to-severe endometriosis (n = 14) exhibited a higher *Shigella/Escherichia* ratio in their colonic microbiota compared to healthy controls (n = 14).

A compromised barrier may facilitate the translocation of *Escherichia coli* (E. coli) from the gut to the pelvic cavity via enterocytes (44). Once in the uterine and peritoneal cavities, LPS binds to TLR4 upon entering the peritoneal fluid. This initiates signalling cascades critical for host immune responses (58). To illustrate, the activation of TLR4 triggers the NF-κB pathway, driving the expression of pro-inflammatory cytokines, including interleukin-6 (IL - 6), tumour necrosis factor-alpha (TNF-α), and IL - 1β, and increasing COX - 2 mediated PGE2 production. This ultimately results in elevated oestrogen synthesis (discussed in section 3.2), ultimately generating a positive feedback loop that further supports lesion survival and growth. A pivotal study by Shan et al, demonstrated that dysbiosis heightened pathways that promoted NF-kB and therefore interleukin-8 (IL - 8) and TNF-α expression; all contributing to an inflammatory response (33). All these play a significant role in inducing endometrial tissue adhesion and angiogenesis along with promoting the formation, infiltration of these endometriosis peritoneal nodules (59–62). Additionally, LPS-TLR4 binding significantly increases immune cell recruitment and alters their functionality, particularly macrophages. This altered macrophage phenotype impairs their phagocytic ability, reducing their capacity to clear newly implanted endometriotic lesions, thereby promoting lesion survival (9, 63, 64). In summary, microbial dysbiosis fosters immune dysfunction in EMS through impaired immune surveillance and increased bacterial proliferation within an inflammatory environment. Insights into these mechanisms through microbial and metabolomics profiling could accelerate the identification of microbial and immune cell biomarkers, enabling the development of non-invasive diagnostic tools and advancing personalised therapeutic strategies.

Beyond direct immune activation, dysbiosis may also disrupt mucosal tolerance mechanisms fundamental to immune homeostasis. The gut-associated lymphoid tissue (GALT), which includes Peyer’s patches, isolated lymphoid follicles and mesenteric lymph nodes; serves as a key component for mucosal immunity. These structures help maintain a delicate balance between immune tolerance towards commensal microbes and dietary antigens, and activation against potential pathogens (65). The gut microbiota plays a pivotal role in educating the immune system by informing T cell development, promoting Treg induction, and guiding pattern recognition receptor (PRR) responsiveness (15). Specific taxa, such as *Bacteroides fragilis* and Clostridia clusters, have been shown to hijack the Treg differentiation process in the gut to promote mucosal tolerance and dampen inflammation (66, 67). In conditions like EMS, dysbiosis may disturb this delicate “training” process, leading to excessive immune activation or impaired immune regulation. Moreover, the reduction in *Firmicutes* (discussed in Section 2.4), key producers of SCFAs, could also impair Treg induction. Since SCFAs are essential for Treg differentiation and immune tolerance, their loss may disrupt Treg function, impairing the immune response, potentially favouring an inflammatory environment which would ultimately contribute to disease progression (68).

### 3.2 The *estrobolome*: hormonal crosstalk between the microbiome and immune regulation

**Figure 4:**
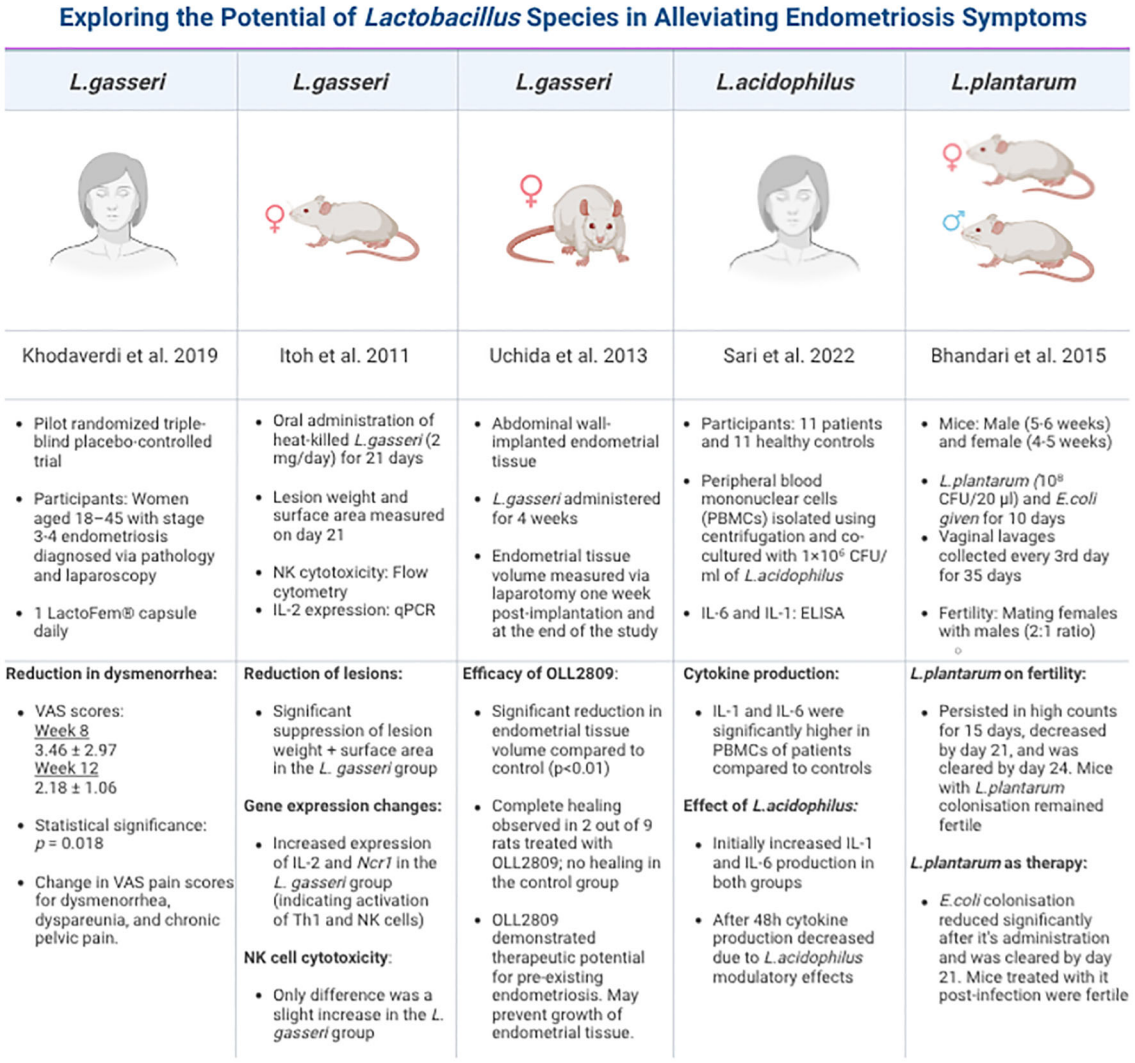
Summary of studies on different *Lactobacillus* species in endometriosis. This table is compiled of pivotal, recent and groundbreaking research that has been conducted using diverse models, strategies and *Lactobacillus* strains. Each study reports the beneficial effects of these strains focusing on their immunomodulatory impact on EMS.

While bacterial contamination and endotoxin-induced immune responses form one axis of immunomodulatory disruption in endometriosis, another critical but often underexplored pathway lies in the interplay between the gut microbiome and oestrogen metabolism; referred to as the *estrobolome* (69). This microbial-hormonal interface provides an additional mechanism through which dysbiosis perpetuates immune dysregulation and chronic inflammation in endometriosis. Throughout a woman’s lifetime, the gut microbiota significantly influences the reproductive endocrine system by interacting with hormones such as oestrogen, which are crucial in immune and metabolic regulation (70). Disequilibrium of oestrogen-modulated pathways has been implicated in the pathophysiology of various female reproductive disorders, including endometriosis (71). Notably, the relationship between oestrogen and the microbiome is bidirectional; while oestrogen levels can shape microbial composition, microbial alterations can, in turn, influence systemic oestrogen levels. For example, GnRH-agonist suppression of oestrogen has been shown to alter uterine microbiota, while oestrogen supplementation promoted *Lactobacillus* dominance in the genital microbiota (72) (**Figure 4**).

The gut microbiome houses the genetic inventory to produce oestrogen-metabolising enzymes; particularly β-glucuronidase and β-glucosidase, through a subset of microbial genes collectively known as the *estrobolome* (69). Bacterial genera such as *Bacteroides*, *Bifidobacterium*, *Escherichia*, and *Lactobacillus* contribute to this enzymatic activity, promoting the deconjugation and reabsorption of oestrogens, thus influencing circulating hormone levels (73, 74). In states of dysbiosis, an increased *Bacillota/Bacteroidota* ratio enhances the abundance of β-glucuronidase-producing bacteria, which in turn raises levels of free oestrogens. This results in amplified oestrogen receptors (ERα and ERβ) signalling and a hyperoestrogenic state; an established feature of endometriosis (75).

The downstream effects of an overactive *estrobolome* contribute to hallmark features of endometriosis, including altered cell proliferation, resistance to apoptosis, increased angiogenesis, and heightened oxidative stress. These changes not only worsen local inflammation but also create an immune environment that supports lesion survival and persistence. Thus, the *estrobolome* represents a key intersection where microbial dysbiosis and hormonal imbalance converge to disrupt immune homeostasis, driving the progression of endometriosis (76).

### 3.3 Impaired immune surveillance and its role in lesion survival in endometriosis

The immune microenvironment of endometriotic lesions is profoundly influenced by chronic exposure to bacterial products, which impairs immune surveillance and promotes lesion survival. This exposure, particularly through LPS-TLR4 signalling, drives a shift in macrophage polarisation toward an immunosuppressive, M2-like phenotype. These pathogenic M2 macrophages, a major cellular component within endometriotic lesions, are found in significantly greater numbers in patients with EMS. Elevated levels of IL - 17A in both plasma and lesions have been shown to stimulate this pathogenic M2 polarisation (77). These alternatively activated (M2) macrophages secrete key immunosuppressive cytokines like IL - 10 and TGF-β, which collectively promote angiogenesis, fibrosis, and immune tolerance (78). This immune deregulation facilitates the escape of ectopic lesions and impairs normal clearance mechanisms, such as NK cell cytotoxicity. While this shift in macrophage phenotype provides a permissive environment for lesion survival and progression, the specific factors influencing M2 polarisation within endometriotic lesions are not yet fully understood. However, M2 macrophage infiltration in ectopic endometrial tissues positively correlates with the expression of markers such as CD47, PDPK1, and LDHA (79). Therefore, designated studies are crucial to fully elucidate the precise molecular mechanisms driving M2 polarisation in endometriosis.

The permissive immune environment of endometriosis is not only defined by macrophage polarisation; it also involves significant alterations in other immune cell populations. To demonstrate, data from a study on EMS patients (n=6-8) showed significantly reduced levels of uterine natural killer (uNK) cells in menstrual effluent (5 – 10%) compared to control subjects (10 – 40%) (p=0.01) (80). Furthermore, in EMS-induced olive baboons (n=8), a notable decrease of approximately 20% in peripheral natural T regulatory cells (nTregs) was detected at 3-month and 9-month intervals, alongside an increase of induced T regulatory cells (iTregs) at the 3-months mark (81). The diminished count of nTregs compromises the system’s capacity to suppress excessive inflammatory responses (82). Considering that the increase in iTregs was generated peripherally, it can be influenced by the inflammatory environment (83). This reflects a shift towards a pro-inflammatory state which is allowed to persist within endometrial lesions due to less effective regulation.

Alongside their altered phenotypes, dysfunctional macrophages in EMS patients are found in significantly greater numbers, facilitating the development of a distinct pro-inflammatory cytokine profile. This includes elevated levels of tumour necrosis factor-alpha (TNF-α), interleukin-8 (IL - 8), interleukin-1 receptor (IL - 1R), vascular endothelial growth factor (VEGF), interleukin-6 (IL - 6), and interleukin-17 (IL - 17). This profile may not only contribute to local and systemic inflammation but also holds promise as an immunological biomarker. In this case, integrating cytokine profiling with metabolomic analyses (discussed in Section 5) could help identify predictive readouts of disease activity or therapeutic responsiveness. A pivotal study by Shan et al, demonstrated that dysbiosis heightened pathways that promoted NF-kB and therefore IL - 8 and TNF-α expression; all contributing to an inflammatory response (33). All these play a significant role in inducing endometrial tissue adhesion and angiogenesis along with promoting the formation, infiltration of these endometriosis peritoneal nodules (59–62). The overall dysregulated immune response is essentially generating an immunosuppressive and inflamed environment; hallmark features of endometriosis facilitating the spread and growth of escaped ectopic endometrial cells outside the uterus (60).

In support, numerous studies have noted evidence of bacterial contamination and elevated inflammatory markers. For example, IL - 17A levels have been shown to positively correlate with the abundance of *Bacteroides* (r = 0.89, p < 0.05) and inversely with *Streptococcus* and *Bifidobacterium* (r = –0.89, p < 0.05) (33). Additional bacterial taxa that exhibited positive correlations include *Actinobacteria*, *Euryarchaeota*, *Fusobacteria*, *Lentisphaerae*, *Spirochaetes*, and *Synergistetes* (81). These microbial shifts are accompanied by immunological changes, including a significant decrease in peripheral natural T regulatory cells (nTregs) at the 3- and 9-month intervals. Conversely, an increase in induced T regulatory cells (iTregs) was observed at 3 months, which showed a negative association with *Porphyromonas* and *Prevotella*. In conjunction, these findings support the presence of a distinct microbiota-immune interaction profile in EMS and suggest that changes in microbial diversity and T cell populations could profoundly affect immune regulation. This strengthens the case for developing non-invasive diagnostic biomarkers based on microbiota and cytokine signatures. However, larger, longitudinal studies are needed to validate these associations and unravel the underlying mechanisms linking the microbiome, immune function, and EMS pathology.

## 4 Current endometriosis landscape: burden, diagnostics, and treatment obstacles

Finding a non-invasive diagnostic biomarker for endometriosis (EMS) is critical due to its profound impact on patients and healthcare systems. EMS significantly impairs physical and mental well-being, leading to higher rates of depression (18.9% vs. 9.3%) and anxiety (29.7% vs. 7.0%) compared to healthy controls (84). Sufferers experience severe menstrual and chronic pelvic pain, alongside common gastrointestinal symptoms like nausea and bloating, affecting 90% of confirmed cases (85–88). Additionally, the economic burden is substantial, with indirect costs in the EU reaching an estimated €54 million annually due to lost workdays (89). Socially, women frequently face minimisation or dismissal of their pain, contributing to an alarming diagnostic delay of 7 to 10 years (90–92). Current diagnostic tools, primarily ultrasound and MRI, can only suggest EMS, not definitively diagnose it (93, 94). Surgical validation is the sole definitive method, yet it’s often inaccessible due to cost and availability, and carries high recurrence rates, with about half of patients needing another surgery within five years, potentially leading to organ deterioration (95, 96). Even after diagnosis, first-line pharmacological treatments like progestins, while versatile, have significant side effects including irregular spotting, mood swings, and weight gain (97). Given the extensive diagnostic delays and adverse treatment effects, there’s a clear, unmet need for personalised diagnostic and clinical approaches to address the heterogeneity of EMS.

## 5 Future directions of endometriosis management

Building on the urgent need for improved strategies, future directions in EMS management are exploring novel, personalised approaches. Emerging research within the field of the gut microbiome suggests that addressing gut dysbiosis and its role in immunomodulation is the key to uncovering the mechanisms underlying the bidirectional relationship between the microbiome and endometriosis. Relevant and significant approaches include the utilisation of probiotics and faecal microbiota transplantation (FMT) which aim to restore eubiosis alongside omics analyses of metabolic derivatives associated with inflammation. These nuanced strategies show great promise in identifying microbial biomarkers for diagnosis and in the provision of personalised therapeutics aimed at alleviating pain, thus paving the way for tailored clinical approaches in the management of endometriosis.

### 5.1 *Lactobacillus-*based probiotics in the treatment of endometriosis

Many researchers have turned to the microbiome in search of answers regarding EMS diagnosis and treatment, spurred by recent findings regarding probiotics’ potential to address a range of diverse health issues from infections and rare genetic disorders to cancer (98, 99). Considering *Lactobacilli* is the most extensively studied probiotic bacteria (100) it was employed by many researchers. For example, both Khodaverdi et al. (101) and Itoh et al. (102) explored the benefits of orally administered *Lactobacillus* (LactoFem^®^) on pain severity in EMS patients through pilot and double-blind placebo-controlled studies; respectively. Their findings concluded that *Lactobacillus gasseri* (OLL2809) was able to ameliorate EMS-associated pain and dysmenorrhea in stage 3 and 4 EMS without any side-effects over an 8-week period. Building on this clinical evidence, Uchida and Kobayashi (103) conducted preclinical trials using a rat model to investigate the biological effects of *L. gasseri* on lesion progression. Their study revealed a statistically significant reduction in EMS lesion size within the abdominal cavity (p < 0.01) and suggested the probiotic’s potential not only for treatment but also for prevention of disease progression, with two rats even demonstrating signs of complete healing (103).

Regarding the immunomodulatory effects of *Lactobacillus* species, a study by Sari et al. (104) investigated this by demonstrating that *L.acidophilus* post 48h of administration, lowered both pro-inflammatory cytokine IL - 6 and IL - 1 concentrations by 29% (104).This suggests probiotics may work by enhancing immunomodulation by increasing both NK cell activity and IL - 12 levels which may counteract the immune dysregulation caused by the gut-dysbiosis (102, 103, 105). Beyond gynaecological pain, probiotics may address EMS-related infertility, with *L.plantarum* showing potential as an infertility therapeutic agent (106). Given EMS patients have a 50% increased risk of developing inflammatory bowel disease (IBD) (107), there is also a fair amount of research suggesting probiotics can alleviate EMS and IBD-related GI symptoms. For instance, a randomised, double-blind study by Weizman et al. (108), looked at 101 paediatric patients with irritable-bowel syndrome (IBS) and revealed that a supplementation of *L. reuteri* (DSM 17938) reduced abdominal pain frequency and intensity in the span of a month (108).

Overall, these findings are impressive however they also highlight the inadequacy of a “one-size-fits-all” approach, as individual efficacy likely varies widely due to the complex interplay of host and microbial factors. Standardised methodologies and large-scale studies encompassing ethnically diverse cohorts are essential for establishing reliable microbial biomarkers. Given the observed variation in EMS immunomodulation across ethnicities, with a higher prevalence reported within Asian women (109), future studies should prioritise underrepresented populations to enhance the generalisability of microbial biomarkers and address health disparities. Beyond ethnicity, factors such as genetics, diet and lifestyle significantly influence microbial profiles suggesting that interventions like probiotics or dietary strategies may require individual tailoring for optimal efficacy. In turn, precision medicine frameworks that integrate host–microbiome interactions offer a promising avenue for developing more targeted and effective treatment strategies for endometriosis.

### 5.2 Conventional and autologous faecal microbiota transplantation in restoring eubiosis

An alternative technology to probiotics, is faecal microbiota transplantation (FMT). It involves delivering stool from a healthy donor to a patient via either enema, colonoscopy or upper GI routes (endoscopy, nasogastric or nasoenteric tubes or oral capsules) (110). FMT’s mechanism of action has been linked to competing with pathogenic bacteria, stimulating the intestinal immune system and protecting the intestinal barrier (111). Maintaining *eubiosis* is imperative for human health and therefore, could be a valuable therapeutic target. Currently, FMT represents the leading innovative technique for accomplishing this (112). The clinical efficacy of FMT has been validated in numerous diseases, to illustrate, a well-documented example is FMT’s use as for recurrent *Clostridioides difficile* infections. Over the last decade, FMT has had a success rate of around 90%, by restoring healthy colonic flora; surpassing the effectiveness of vancomycin (113).

To date, there are currently no clinical reports that outline FMT application in gynaecological disorders except in mouse models. However, laboratory research data provides a solid foundation to encourage further studies involving human models. For instance, a study conducted by Kim et al. (114) displayed that administering NK49 (*B.longum*) and NK3 (*L.plantarum*) individually and combined, reduced GV-induced BV in mice. Supporting this observation was a decrease in TNF-a levels and *Pseudomonadota* alongside an increase in IL - 10 and *Bacteroidota.* These bacterial-induced changes inhibited LPS production by the gut microbiota, partially “reversing” inflammation through induced immunomodulation (114).These findings suggest a novel and effective approach for treating endometriosis or at least reducing its symptoms by targeting microbiome restoration.

However, FMT’s risks and challenges must be accounted for as well. A successful FMT requires strict donor selection that excludes immunocompromised or comorbid patients, fresh treatment preparation and pathogen screening. Satisfying all these criteria proves challenging in both logistic and financial aspects (115). Continuing, FMT also poses some clinical risks. For instance, transplantation of disease-associated microbiota has been seen to trigger pathology such as diarrhoea, abdominal cramping and nausea (116). The possibility of long-term adverse effects to patients due to the alteration of their gut microbiota is another concern. In an effort to overcome conventional FMT’s limitations, autologous FMT (aFMT) has emerged. In aFMT, a patient’s own microbiome is collected during a healthy state and later reintroduced when illness occurs. This could eliminate extremely selective processes and enhance long-term sustainability (117). Despite its encouraging prospects, further extensive research is necessary to validate the safety, efficacy, long-term outcomes, cost-effectiveness and affordability of this microbiome-targeted intervention in EMS, before it’s implemented in a clinical setting.

### 5.3 Metabolomics profiling in the development of microbial biomarkers

In parallel with mechanistic-focused research, the incorporation of advanced omics techniques, particularly metabolomics should be a priority in future investigations. Metabolomics refers to the analysis of small-molecule metabolites in tissues or biofluids, providing insights into the physiological or pathological state of a system (118). Its recent application in cancer and chronic inflammatory diseases has proven valuable in identifying biomarkers, such as specific levels and types of short-chain fatty acids (SCFAs), which are microbial-derived metabolites that modulate host immunity (119–121). In the context of EMS, metabolomic approaches have begun to uncover characteristic signatures. For instance, Ni et al. (48) reported altered faecal metabolites related to secondary bile acid biosynthesis in murine models, with decreased levels of alpha-linoleic acid (ALA); a compound known for its intestinal protective and anti-inflammatory roles (122). Similarly, a systematic review by Adamyan et al. (123) highlighted increased levels of succinate, β-hydroxybutyric acid, and ketone bodies in EMS patient biofluids, linking metabolic dysregulation to disease pathology and oxidative stress (123).

To complement these metabolic markers, emerging evidence supports the use of immune cell-derived cytokines such as IL - 17A, IL - 6, and TNF-α as immunological biomarkers of dysbiosis. These pro-inflammatory cytokines are elevated in EMS and are closely tied to microbial imbalances, especially in the gut and reproductive tract. IL - 17A, for example, shows strong correlations with specific microbial taxa such as Bacteroides and is known to drive inflammatory tissue responses (33). When analysed alongside metabolomic profiles, such immune mediators could serve as functional readouts of microbial activity and host response, strengthening biomarker precision and interpretability. Together, the integration of metabolomics with immunological biomarkers offers a powerful, non-invasive approach for monitoring disease progression, stratifying patients by inflammatory or metabolic subtypes, and enabling more personalised therapeutic strategies. Future research should prioritise the co-analysis of microbiota composition, metabolite signatures, and cytokine profiles, ideally using longitudinal cohort designs and high-resolution multi-omics platforms.

Metabolomics holds vast potential for non-invasive and precise detection of immunomodulatory disruptions caused by EMS. This innovative approach could be leveraged to monitor disease progression, stratify patients, and enable personalised treatments tailored to individual metabolomic profiles.

## 6 Conclusion

Gut dysbiosis is increasingly recognised as a key factor in the pathogenesis of EMS, particularly through its dysregulation of key processes within the female reproductive system. These include the modulation of oestrogen pathways, metabolic derivatives, oxidative stress, and immune-mediated inflammation. This paper specifically focuses on the dysregulation of immunomodulation and its role in driving immune dysfunction and perpetuating chronic inflammation. Underlying this immune imbalance is the disruption of mucosal immune structures (e.g GALT and Peyer’s patches), which normally support immune tolerance by mediating the microbiome’s regulation of innate and adaptive immunity. Although microbiome-based therapies, such as probiotics and FMT hold considerable promise, their clinical implementation remains halted by unresolved questions regarding microbial diversity, safety, and efficacy. Progress in this field will require the integration of advanced omics technologies, large-scale cohort studies, and efforts to address ethical, genetic, diversity-related, and economic barriers. An extensive understanding of the connection between gut microbiota alterations to endometriosis is necessary. It could comprehensively map these pathways and tackle gaps in interpreting and translating gut microbiota findings, informing early diagnosis and targeted interventions in a non-invasive manner. Moreover, it could support the development of personalised therapeutic approaches, tailored to unique microbiome profiles and genetic predispositions of individual patients. However, achieving this will require collaborative research efforts to bridge the gap between scientific discovery and clinical application; this represents the critical key to unlocking the untapped potential of the gut microbiome in the care and management of endometriosis.
